# Labdanum Resin from *Cistus ladanifer* L.: A Natural and Sustainable Ingredient for Skin Care Cosmetics with Relevant Cosmeceutical Bioactivities

**DOI:** 10.3390/plants11111477

**Published:** 2022-05-31

**Authors:** David F. Frazão, Carlos Martins-Gomes, Jan L. Steck, Judith Keller, Fernanda Delgado, José C. Gonçalves, Mirko Bunzel, Cristina M. B. S. Pintado, Teresa Sosa Díaz, Amélia M. Silva

**Affiliations:** 1 Center for Research and Technology of Agro-Environmental and Biological Sciences (CITAB-UTAD), University of Trás-os-Montes e Alto Douro (UTAD), Quinta de Prados, 5001-801 Vila Real, Portugal; davidmfrazao@gmail.com (D.F.F.); camgomes@utad.pt (C.M.-G.); 2 Plant Biotechnology Center of Beira Interior (CBPBI), Quinta da Senhora de Mércules, Apartado 119, 6001-909 Castelo Branco, Portugal; fdelgado@ipcb.pt (F.D.); jcgoncalves@ipcb.pt (J.C.G.); cpintado@ipcb.pt (C.M.B.S.P.); 3 Department of Food Chemistry and Phytochemistry, Institute of Applied Biosciences, Karlsruhe Institute of Technology (KIT), Adenauerring 20a, Building 50.41, 76131 Karlsruhe, Germany; jan.steck@kit.edu (J.L.S.); judith.keller@kit.edu (J.K.); mirko.bunzel@kit.edu (M.B.); 4 Polytechnic Institute of Castelo Branco-School of Agriculture (IPCB-ESA), Quinta da Senhora de Mércules, 6001-909 Castelo Branco, Portugal; 5 Department of Plant Biology, Ecology and Earth Sciences, Faculty of Science, University of Extremadura, 06006 Badajoz, Spain; tesosa@unex.es; 6 Department of Biology and Environment, School of Life Sciences and Environment, UTAD, Quinta de Prados, 5001-801 Vila Real, Portugal

**Keywords:** *Cistus landanifer* L., anti-inflammatory, antimicrobial, antioxidant, diterpene, flavonoid, sunscreen effect, rockrose

## Abstract

Labdanum resin from *Cistus ladanifer* L. (Cistaceae) is an abundant natural resource in the Iberian Peninsula worth being explored in a sustainable manner. It is already used in the cosmetic industry; mainly by the fragrances/perfumery sector. However, given the highest market share and traditional uses, labdanum resin also has the potential to be used and valued as a cosmetic ingredient for skincare. Aiming to evaluate this potential, labdanum methanolic absolute and fractions purified by column chromatography were characterized by UPLC-DAD-ESI-MS and then evaluated for UV-protection, antioxidant, anti-elastase, anti-inflammatory, and antimicrobial activities. Labdanum absolute represented ~70% of the resin; diterpenoid and flavonoid fractions represented ~75% and 15% of the absolute, respectively. Labdane-type diterpenoids and methylated flavonoids were the main compounds in labdanum absolute and in diterpenoid and flavonoid fractions, respectively. Labdanum absolute showed a spectrophotometric sun protection factor (SPF) near 5, which is mainly due to flavonoids, as the flavonoids’ SPF was 13. Low antioxidant activity was observed, with ABTS radical scavenging being the most significant (0.142 ± 0.017, 0.379 ± 0.039 and 0.010 ± 0.003 mgTE/mgExt, for the absolute and flavonoid and terpene fractions, respectively). Anti-aging and anti-inflammatory activity are reported here for the first time, by the inhibition of elastase activity (22% and 13%, by absolute and flavonoid extract at 1 mg/mL), and by the inhibition of nitric oxide production in LPS-induced RAW 264.7 cells (84% to 98%, at 15 µg/mL extracts, flavonoid fraction the most active), respectively. Antimicrobial activity, against relevant skin and cosmetic product microorganisms, *Staphylococcus aureus, Pseudomonas aeruginosa*, *Candida albicans*, and *Escherichia coli*, revealed that only *S. aureus* was susceptible to labdanum absolute (MIC: 1.2 mg/mL) and its fractions (MIC: <0.3 mg/mL). In conclusion, labdanum resin showed potential to be used in sunscreen cosmetics, anti-inflammatory skincare cosmeceuticals or medicines but has low potential as a cosmetic product preservative given the low antioxidant and low-spectrum antimicrobial activities.

## 1. Introduction

The rockrose *Cistus ladanifer* L. (Cistaceae) is an abundant and widespread plant resource in the Iberian Peninsula, also present in the South of France and North Africa [1,2]. From its photosynthetic leaves and stems [3], *C. ladanifer* exudes the commonly named “labdanum gum”, which is in fact a resin instead of a gum [4]. Industrially and traditionally, this resin is usually extracted from the plant material with alkaline water followed by acid neutralization, but it may also be extracted using just boiling water but with lower extraction yields [5,6,7]. This resin is a complex mixture of terpenoid and phenolic compounds, which are represented mainly by labdane-type diterpenes and methylated flavonoids [3,8]. Although waxes may not be considered constituents of the resin, they are extracted together either with the alkaline water process [6] or with organic solvents, such as chloroform [3]. To remove waxes, labdanum must be dissolved in warm methanol and then cooled down to negative temperatures to precipitate and separate, by centrifugation, the waxes [6,7,9,10], rendering the labdanum absolute. Labdanum absolute may be separated into diterpenoid and flavonoid fractions by molecular sizing column chromatography [3,10,11,12].

A current application of labdanum resin and its extracts is in the perfumery/fragrance industry [13]. The global market for cosmetics in 2020 was estimated to be higher than 200 billion euros [14]. Although fragrances/perfumery products have a significant market share, skincare products dominate the cosmetics market [14,15]. Interestingly, labdanum resin is documented to be traditionally used on human and animal skin to treat wounds [16]. In addition, labdanum resin and labdanum absolute are already registered in the European Chemicals Agency (ECHA) according to Regulation (CE) 1907/2006, which establishes a registration system for chemical products (REACH) to be used or traded within the European Union. In fact, those registrations predict the end use of the “substances” in fragrance and cosmetic products, between other uses. A consumer-driven trend for cosmetic products made from natural and sustainable ingredients [17,18] justifies even more the evaluation of labdanum resin/absolute as an ingredient for skin care cosmetics.

Skin topical cosmetics are mainly used to restore or maintain skin barrier properties by including moisturizers, antioxidants, anti-inflammatory agents, or even UV-protection agents (Pilae et al., 2016). Skin exposure to UV radiation leads mainly to skin photoaging damage (premature skin aging) but can also result in erythema (sunburn), immunosuppression, and carcinogenesis [19]. Given the harmful effects of such radiation on the skin, the use of sunscreens is widely advised [20]. Sun Protection Factor (SPF) is a measure of sunscreen protection against UVB (280–320 nm) radiation, based on erythema symptoms, and currently only in vivo methods are accepted to determine it [21]. However, for prospection purposes, a simple spectrophotometric method for SPF determination was proposed by Mansur, et al. [22] and implemented by Dutra, et al. [23] and Gaweł-Bęben, et al. [24].

Reactive Oxygen Species (ROS) generation by both UVA and UVB radiation is a known mechanism that leads to skin damage by direct chemical modification of nuclear and mitochondrial DNA, cell lipids, and dermal matrix proteins, such as collagen produced by dermal fibroblasts, or damage of the cornified envelop in the epidermis *stratum corneum* that functions as a permeability and mechanical barrier [19]. Antioxidants can scavenge ROS to prevent the harmful effects of oxidative stress on the skin. In addition, antioxidant ingredients in a cosmetic contribute to the extent of its shelf-life. There are several methods to evaluate the antioxidant activity of substances or mixtures, some based on the transfer of hydrogen atoms (ORAC test), others based on the transfer of electrons (FRAP and Folin–Ciocalteu tests) and also based on the transfer of hydrogen atoms and/or electrons (ABTS and DPPH tests) [25].

The activation of transduction pathways directly by UVB radiation compiles a general mechanism that leads to skin damage [19]. A variety of pro-inflammatory mediators are induced by UVB radiation [26]. Sunburns induced by UVB radiation are characterized by acute inflammatory responses causing skin structure damage [26]. In addition, UV-induced chronic inflammation is associated with cancer development [27]. Macrophages are crucial for the inflammatory response and once stimulated, produce pro-inflammatory mediators (e.g., cytokines, nitric oxide) and have been extensively used to evaluate the anti-inflammatory activity of various substances [28].

Elastases are enzymes responsible for the breakdown of elastin and fibronectin, and other proteins responsible for the elasticity of connective tissue [29]. Under physiological conditions the activity of elastase is controlled (inhibited) by serpins; however, this balance can be disturbed by several mechanisms, such as ROS, and UV-radiation, leading to skin barrier disturbance and contributing to skin inflammation, loss of skin elasticity and wrinkling formation [30]. Compounds capable of inhibiting elastase activity would, therefore, have a beneficial property for cosmetic skincare ingredients, preventing an additional pathway of skin aging.

Skin microflora (or skin microbiome) are important to prevent, but may also contribute to, skin diseases. Gram-positive bacteria staphylococci, in particular, *Staphylococcus aureus* and *Staphylococcus epidermis* are well-known and prominent opportunistic microorganisms that cause skin infection, inhibiting healing and promoting the further inflammation of skin lesions [31,32]. Gram-negative bacteria *Pseudomonas aeruginosa* [33] and the yeast *Candida albicans* [34] are also recognized as skin colonizers and opportunistic pathogens, although are not so frequent. Cosmetic ingredients with antimicrobial activity may thus help to prevent skin infections and also, like antioxidants, increase the final product shelf-life. In fact, according to European Standard EN ISO 17516:2014, the microbiological quality/safety of a cosmetic must be evaluated regarding total aerobic mesophilic microorganisms, *Escherichia coli*, *S. aureus*, *P. aeruginosa* and *C. albicans*. 

Thus, responding to the high demand for using natural-based products and using alternative sources under a circular economy approach, the main aim of this study is to evaluate the potential of labdanum resin as a natural and sustainable ingredient for skincare cosmetics, by evaluating its ability in maintaining skin health, through the evaluation of labdanum resin’s anti-oxidant, anti-inflammatory and UV-protection potential, and also by exploring its antimicrobial activities in order to promote skincare, but also to act as a preservative in the final cosmetic product.

## 2. Results and discussion

### 2.1. Extraction and Yields of Labdanum Absolute and Its Fractions

In this study, labdanum resin was extracted using the “Andalusian process”, yielding 7.44 ± 0.41% (DW/FW) of labdanum resin in relation to plant material (Table 1). The “Andalusian process” was chosen in contrast to the “Zamorean process” because of its higher resin yields [6]. The difference between the two methods is that the latter uses boiling water to physically remove, by melting, the resin from the plant material instead of the alkalinization and, when cooled down, the resin precipitates. The yield obtained in this study is similar to the one obtained by Burguer [6]. In fact, the same process was used to extract the resin, only differing in the workup processes, such as centrifugation, and the final freeze-drying step. The Na_2_CO_3_ solution (25 g/L; 0.24 M) was considered a buffer solution as it was able to keep the pH at approximately 11 during the extraction. Greche, et al. [7] used a similar process because albeit using a lower concentration of NaHCO_3_ (0.1 M), which is about half the one used in this study, they used a lower proportion of fresh plant material in relation to the extraction solution, 0.075 g/L, compared to the 0.125 g/L used in this study. Greche, et al. [7] did not report resin yields, but the extraction process differs in this study; the acidification was conducted until pH 2 instead of neutralizing the solution. However, carbonate neutralization was attempted in this study but it was further excluded because (i) no specific pH could be reached (i.e., pH was unstable after carbonate neutralization), (ii) after neutralization, albeit obtaining a precipitate, the supernatant was not as clear as the one obtained at pH 2, and (iii) the volume of acid used between neutralization and acidification, to reach pH 2, was little (~1–1.5 mL) compared to the volume needed for the neutralization (~6–7 mL) of initial extract (at pH 11).

According to Handa [35], a resinoid is defined as the extract obtained from a natural resin using a hydrocarbon solvent. An absolute is an alcohol extract of a hydrocarbon extract, usually conducted at 40–60 °C and then cooled to negative temperatures, to separate waxes. Among the literature, these terms are not consistent. For instance, Greche, et al. [7] named an ethanol extract from the resin a resinoid. In this study, labdanum absolute is considered the best term for the direct methanolic extract of crude labdanum resin. The yield of labdanum absolute was 73.3 ± 0.9% (DW/DW) from labdanum resin (Table 1). According to Burguer [6], methanol is the best solvent to separate the long chain hydrocarbons from the crude resin compared to hexane or ethanol, obtaining a methanol absolute yield of 62.3% (DW/DW), a value slightly lower than the one here obtained (Table 1). Other authors also used methanol to separate waxes from chloroform extracts of the plant exudate [3,10,12].

Fractionation of labdanum absolute by molecular sizing column chromatography (Table 1), rendered the diterpenoid fraction as the major fraction (~75% DW/DW) which was eluted first followed by the flavonoid fraction with a yield of around 15% (DW/DW). The remainder, calculated by difference, accounted for up to 10% (DW/DW). A part of the remainder was collected after flavonoids, during a long period and with a large amount of eluent, recovering only 6.72 ± 0.79% (DW/DW) of the absolute. According to Chaves, et al. [36] and Sosa, et al. [10] flavonoids account for between 14 to 28% (DW/DW) of the resin, for plants harvested in summer, and between 6 to 16% (DW/DW), for plants harvested in winter, which is in accordance with the fraction yield obtained in this study (plants harvested in summer, see methods). According to Alías et al. (2012), 6-oxocativic acid, 7-oxo-labd-8(9)-en-15-oic acid, and 6β-acetoxy-7-oxo- labd-8(9)-en-15-oic acid, together, accounted to 21 mg/g (DW/DW) of mature leaves in winter and dropped to approximately 10 mg/g (DW/DW) in the other seasons. Considering the exudate yields from Sosa, et al. [10], these three diterpenes alone would account for between 6 to 16% (DW/DW) of the resin which is far below the diterpenoid fraction yield obtained in this study. However, labdanum resin is known to be composed of more labdane-type diterpenes, such as labdanolic acid [7,37]. Labdanolic acid is a major compound in labdanum resin as shown by Alías [8], Greche, et al. [7], and Burguer [6]; therefore, the diterpenoid yield in labdanum resin of 6–16% (DW/DW) might be underestimated.

### 2.2. Chemical Profile of Labdanum Absolute and of Its Fractions

The chemical profile of labdanum absolute and fractions is presented in Table 2, which summarizes the information obtained by UPLC-DAD-ESI-MS analysis. Chromatograms of labdanum absolute and its fractions are presented in the supplementary material (Figure S1). The three extracts present distinct chromatographic profiles which, in turn, confirmed the composition of the purified fractions. 

The chromatogram of the flavonoid fraction revealed seven peaks (Figure S1.3 and Table 2) that could be ascribed to flavonoids described in the literature, namely apigenin-4′-methyl ether (peak 3, 24.7 min) and apigenin-7-methyl ether (peak 4, 24.9 min) were distinguished based on their order of elution from other reversed-phase chromatographic methods [3]. Despite two different kaempferol-dimethyl ethers being described in the literature, only one peak (5, 26.6 min) was observed that could be assigned to both compounds; however, kaempferol-3,7-methyl ether (jaranol) is more frequently reported in the literature, in addition to being reported as a major flavonoid in labdanum resin [10]. 

Diterpene fraction revealed 14 peaks in the chromatogram (Figure S1.2 and Table 2) but only five could be assigned to compounds described in the literature. All of those matches are labdane-type diterpenoids, which have also been shown to be a major group of compounds in labdanum resin [7,12,37,49]. Peaks 13 (35.4 min) and 15 (37.4 min) were the major peaks in the diterpenoid fraction chromatogram and were assigned to compounds already described as major diterpenoid compounds, oxo-labdenoic acid (most likely 6-oxo-cativic acid) and labdanolic acid, respectively. Labdanolic acid does not absorb UV radiation and because of that, it cannot be detected using classical HPLC methods using DAD or UV detectors. 

The labdanum absolute (Figure S1.1 and Table 2) chromatogram showed a chemical profile in which the same flavonoids present in the flavonoid fraction were predominantly detected but also showed some major non-identified peaks of compounds with longer retention times, such as peak 20 (42.3 min) and peak 23 (45.9 min). These non-identified compounds were lost when obtaining the purified diterpenoid and flavonoid fractions by molecular sizing chromatography. Some diterpenes were detected as lower intensity peaks and interestingly, the major peak of the diterpene fraction (labdanolic acid, peak 15, 37.4 min) was not detected in the absolute chromatogram. 

### 2.3. Assessment of Labdanum Absolute and Labdanum Fractions Sun Protection Factor

**Figure 1:**
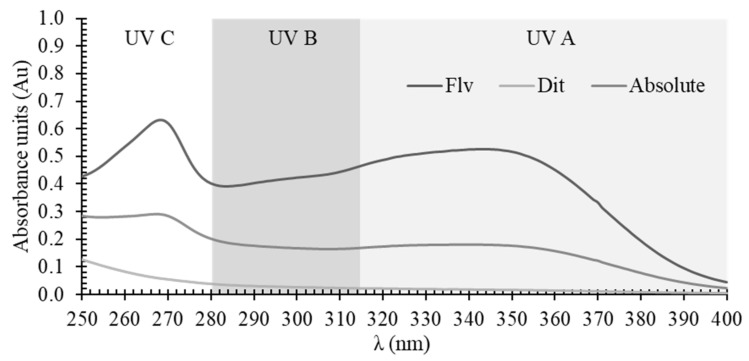
UV absorption (250–400 nm) spectral scan of labdanum absolute, flavonoid fraction (Flv), and diterpene fraction (Dit) related to a concentration of 100 µg/mL (*n* = 3, mean curve).

Sun Protection Factor (SPF), as well as the integral of the absorption spectra at UVB (280–315 nm) and UVA (315–400 nm) regions of labdanum absolute, the diterpenoid fraction, and flavonoid fraction are presented in Table 3. The UV absorption spectra of the extracts are shown in Figure 1. Regarding all three parameters, the flavonoid fraction showed the highest values followed by labdanum absolute. The diterpenoid fraction showed very low UV absorption. Labdanum absolute showed a UVB SPF value (4.98 ± 0.19) that is very similar to the values obtained by Gaweł-Bęben, et al. [24] for *C. ladanifer* methanolic extracts (between 3.33 ± 0.15 and 4.37 ± 0.42). In this work, we further demonstrate the significant absorbance of UVA radiation by labdanum absolute and that flavonoids are responsible for that feature, whereas the diterpenoid fraction contribution is very low or even negligible; this directly correlates with the chemical structures of the compound classes. Our results show that, at least partially due to flavonoids, labdanum absolute possesses a broad spectrum of UV protection by absorption, and therefore, labdanum resin may be considered a natural ingredient with the potential to be included in sunscreen formulations. 

### 2.4. Antioxidant Activity of Labdanum Resin and Its Fractions

Antioxidant activity results of labdanum absolute and its purified fractions are presented in Table 4. Results are expressed as equivalents to the well-known antioxidant compounds, gallic acid and Trolox. DPPH and FRAP methods showed a very low antioxidant activity in all the extracts in relation to the Trolox standard. For these two methods, all the extracts presented values below 0.1 mgTE/mgExt (i.e., 0.4 mmolTE/gExt) meaning that they have an antioxidant activity more than 10 times lower than the standard. In contrast, ABTS and Folin–Ciocalteu methods showed more prominent antioxidant activity in relation to Trolox and gallic acid, respectively, for labdanum absolute and flavonoid fraction extracts. The flavonoid fraction showed an ABTS antioxidant activity around three times lower than Trolox and a Folin–Ciocalteu antioxidant activity around five times lower than gallic acid. Labdanum absolute showed an ABTS and Folin–Ciocalteu antioxidant activity less than 10 times lower than the standards. Despite the differences between methods, the labdanum absolute and flavonoid fraction antioxidant activities were not statistically different, in each of the methods used (Table 4). 

Labdanum absolute has fewer flavonoids than the purified flavonoid fraction. This would mean that although flavonoids contribute to the antioxidant activity of the labdanum absolute, other compounds in the labdanum absolute not included in the diterpenoid fraction must also contribute to the antioxidant activity. In fact, labdanum absolute was shown to have significant compounds not present in both the purified fractions but that could not be assigned to a known component of the labdanum resin described in the literature (Table 2). *C. ladanifer* methanol extraction conducted by Chaves, et al. [50] contained a lower Folin–Ciocalteu antioxidant activity than the labdanum absolute of this study, but according to the authors, among different plant species in the Iberian Peninsula, *C. ladanifer* is in the group of plants with higher antioxidant activity. In sum, the in vitro antioxidant activity of the labdanum resin is demonstrated, which seems to be related to the typical flavonoids of the resin but not exclusively. This property may confer an additional activity to the potential ingredients to be used in skincare cosmetics, conferring antioxidant activity and thus mitigating/scavenging ROS generation, which can be induced by several factors, such as UV radiation, causing skin damage or photo-aging. Other potential applications include the use of these ingredients as preservatives in cosmetic products. 

### 2.5. Anti-Elastase Activity

Elastase inhibition was observed only for labdanum absolute at the highest concentration used, 1 mg/mL (Table 5). At 0.5 mg/mL none of the extracts showed elastase inhibition activity. Inhibition of elastase activity is shown for several Mediterranean plants, including representatives of *Cistus* spp. [51]. Inhibition of elastase activity and thus the breakdown of skin elastin and other proteins responsible for connective tissue elasticity reduces the skin aging process and would be an interesting property for a skincare cosmetic ingredient. In this study, the weak inhibition activity regarding this enzyme by labdanum absolute is demonstrated. To the best of our knowledge, this study is the first to report the elastase-inhibiting activity of *C. ladanifer* labdanum. However, Gaweł-Bęben, et al. [24] demonstrated that *C. ladanifer* methanolic extracts inhibited, to some extent, tyrosinase activity, which is a target enzyme in the treatment of hyperpigmentation, a result of skin photo-aging.

### 2.6. Anti-Inflammatory Activity

The anti-inflammatory activity of labdanum absolute and fractions was assessed based on the quantification of the pro-inflammatory mediator, nitric oxide (NO), released by lipopolysaccharide (LPS)-stimulated RAW 264.7 cells. By binding to Toll-like Receptor-4 (TLR4), LPS activates intracellular cascades that lead to the expression of inducible nitric oxide synthase (iNOS) which produces NO from L-arginine [52,53]. TLRs are a large family of receptors present at the cell membrane of many cells, including macrophages and many types of skin cells, that respond to pathogen’s components (e.g., LPS) but also to products of host damaged cells [53]. On the other hand, NO has a relevant role in regulating cutaneous inflammation and high levels of iNOS and endothelial-NOS (eNOS) have been implicated in skin inflammatory diseases [54]. Moreover, macrophages play a crucial role in maintaining and restoring skin homeostasis through wound repair, cancer defense and other relevant mechanisms [55]. Thus, studying the effect of labdanum resin extracts in modulating NO release from macrophage cells is relevant to assessing the anti-inflammatory effects of products intended to be used in skincare products.

**Figure 2:**
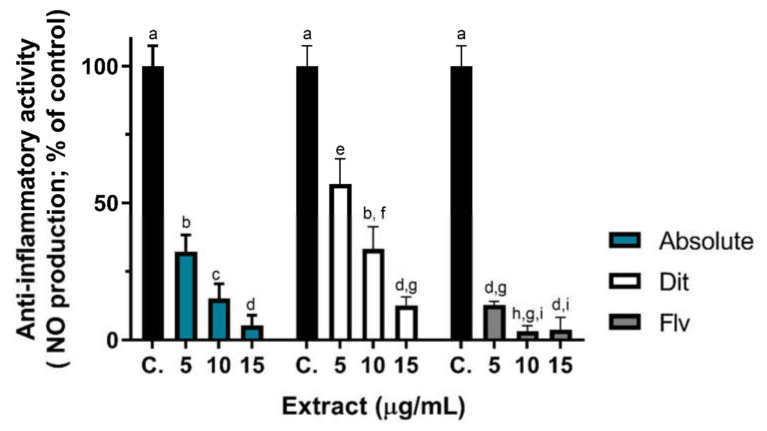
Anti-inflammatory activity of labdanum absolute and fractions. Inhibition of nitric oxide (NO) release by LPS-stimulated RAW 264.7 cells for cells exposed to labdanum absolute (left bars, blue color) and diterpene (Dit, middle bars, white color) and flavonoid (Flv, right bars; grey color) fractions, expressed as percentage of control (black bars). Results are expressed as mean ± SD (*n* = 4 independent assays). Different coefficient letters mean statistical differences between values by the post-hoc Dunnett’s T3 test or Welch’s *t*-test (*p* < 0.05).

The concentrations of extracts to be tested were selected based on RAW 264.7 cells viability assay results (supplementary material Figure S2), and only non-cytotoxic concentrations were tested (concentrations up to 15 µg/mL), and the respective results are presented in Figure 2. The flavonoid fraction (Figure 2, grey bars) produced the highest anti-inflammatory activity in a concentration dependent manner, inhibiting near 90% of NO release in relation to the control, at the lowest tested concentration (5 µg/mL). At 15 µg/mL, the flavonoid and absolute fraction anti-inflammatory activities were similar (*p* < 0.05), reaching near 98% inhibition of NO release. Both extracts showed a concentration dependent activity. Although the diterpene fraction, at 25 µg/mL slightly affected cell viability (at 25 µg/mL, cell viability was ~80% of control; Figure S2), and at 12.5 µg/mL cell viability was ~95% of control, we present the NO production by LPS-stimulated cells exposed to 10 and 15 µg/mL of diterpene fraction, which was on average, higher than that produced by exposure to the other extracts (at the same concentrations), indicating lower anti-inflammatory action (Figure 2). However, compared with control cells, the labdanum resin diterpene fraction showed significant anti-inflammatory activity at the lowest tested concentration (5 µg/mL), reducing ~40% of NO release (Figure 2, white bars) and at this concentration RAW 264.7 cells show 100% viability (Figure S2). In sum, labdanum absolute presents anti-inflammatory activity, a property given prominently by flavonoids (the most active fraction) but also by some components of the diterpenoid fraction (Figure 2). 

To the best of our knowledge, this is the first work reporting the in vitro anti-inflammatory activity of the labdanum resin (and of its fractions). Nevertheless, Youbi, et al. [56] reported the anti-inflammatory effect of *C. ladanifer* aqueous extracts in an in vivo rat model of inflammation (Wistar rats; carrageenan-induced paw edema). Phenolic rich extracts of other Portuguese Mediterranean plants, i.e., *Thymus zygis* subsp. *zygis*, showed NO release inhibition of 48% and of 89%, by aqueous and hydroethanolic extracts at 50 µg/mL, respectively [28], using the same testing method. However, when looking at the results of the absolute and flavonoid fraction at 5µg/mL (Figure 2) the same NO release inhibition was observed but with a ten-fold lower concentration, indicating that labdanum resin has high anti-inflammatory activity. In addition to the SPF, antioxidant, and elastase inhibition activities, the anti-inflammatory activity may give an added-value to the labdanum resin as a cosmetic ingredient, worth incorporating in skin care products since inflammation (erythema) is a UV-mediated skin damage effect as discussed earlier. 

### 2.7. Antimicrobial Activity

Antimicrobial activity results of labdanum absolute and fractions against reference strains relevant for skincare and cosmetics are shown in Table 6. Labdanum absolute and fractions were only active against the Gram-positive bacteria *S. aureus* ATCC^®^ 25923^TM^, a clinical isolate indicated for infectious diseases research. Labdanum absolute showed *S. aureus* inhibition at 1.2 mg/mL (the MIC value); however, the purified fractions showed a much lower MIC value (<300 µg/mL), indicating that the diterpenes and the flavonoids are the main compounds responsible for the *S. aureus* growth inhibitory activity. Concerning the bactericidal activity, the diterpene and flavonoid fraction show a bactericidal activity at 2.5 mg/mL, and the labdanum absolute MMC could not show this activity with the range of concentrations tested. We may assume that the components of each purified fraction are the ones with higher antimicrobial activity; the other components (other than diterpenes and flavonoids) in the absolute may have antagonism or no antimicrobial activity, which highly reduces the labdanum absolute MIC and MMC activity. To our knowledge, only two works report the antimicrobial activity of labdanum resin, the ones conducted by Greche, et al. [7] and by Rauwald, et al. [57]. Rauwald, et al. [57] tested growth inhibition of *Borrelia burgdorferi* and showed no susceptibility for labdanum resins from *C. ladanifer* (at 0.2 mg/mL). Greche et al. (2009) showed broad-spectrum growth inhibition for labdanum absolute and similar extracts (concrete and resinoid), at concentrations between 1 and 10 mg/mL. Labdanum absolute showed growth inhibitory activity against strains also tested in this work, however with a higher MIC, for example, *S. aureus* (MIC: 2–4 mg/mL) and *E. coli* (MIC: >5 mg/mL), but no microbicide activity of the absolute, for any of the tested microorganisms, was observed for the higher concentrations (10 mg/mL) [7]. Thus, the labdanum absolute extracted in this work has higher antimicrobial activity, and it can be potentiated by fractionating it into terpenoid and flavonoid fractions (Table 6). Excluding essential oils, several works evaluating the antimicrobial activity of *C. ladanifer* extracts are published. However, those extracts usually use milled plants and the whole extract is tested which makes them different from the labdanum resin here studied. Boy, et al. [58] showed a broad-spectrum antimicrobial activity, by disk diffusion, of 90% ethanol (*v/v*) extracts obtained from *C. ladanifer* intact leaves (a phenolic rich extract), at concentrations comparable to this study (0.5–2 mg/mL). In fact, other works showed *P. aeruginosa*, *S. aureus, E. coli,* and *Candida* spp. susceptibility for ethanolic/aqueous extracts of *C. ladanifer* rich in phenolic acids and tannins [59,60,61,62]. Methanol, ethanol, and acetone/water extracts from milled *C. ladanifer* material are also reported to have a broad-antimicrobial activity, however, higher in Gram-positive bacteria [63,64]. Comparing our results to the literature leads to the hypothesis that terpenes, flavonoids, tannins and other phenolic compounds are the compounds responsible for the broad-spectrum antimicrobial activity found for *C. ladanifer* extracts. During labdanum extraction, some of those compounds most probably are retained in the “residual” water of the extraction process because of their hydrophilic character or are extracted at low amounts compared to other components.

The four microorganisms tested in this study are important regarding the quality and safety microbiological criteria for final cosmetic products, which must be absent in 1 mg or mL of product according to the European Commission guidelines. In addition, *Staphylococcus aureus, P. aeruginosa*, and *C. albicans* are important because they can cause opportunistic skin infections. Although labdanum resin does not show a high potential preservative application, the high inhibition susceptibility shown against *S aureus* is an important property because it causes the most frequent skin opportunistic infection, as discussed earlier.

Within the European Union, Regulation (EC) nº 1223/2009 establishes standard rules for cosmetic products. Although the cosmetics regulation of the European Commission allows some active ingredients (e.g., sunscreens) to “protect or keep the human body in good conditions” if those ingredients are used to “restore, correct or modify physiological functions” by means of a “pharmacological, immunological or metabolic action” the final product must be considered medicinal. It is thus a question of the intent of the product in question, however, Directive 2001/83/EC clearly states that when in doubt regarding definitions, the product would be considered a medicinal product. In fact, there is no clear official definition and regulation in the world for cosmeceuticals which would be products that are between cosmetic and pharmaceutical/medicinal products [65]. Elastase inhibition and anti-inflammatory activities would be properties to classify a skincare product as a medicinal product. UV radiation protection/filter ingredients may be included in cosmetics according to EU regulation but must be listed in annex IV of the regulation and used according to specifications. In addition, a complete toxicological profile of cosmetic ingredients according to the regulation is also mandatory, particularly photo-induced toxicity, for UV filter substances. There are three ECHA registrations (REACH regulation) regarding labdanum resin with a complete chemical safety assessment: (i) “Gum of *Cistus ladaniferus* (Cistaceae) obtained from stems and leaves by extraction with alkaline solution”, raw labdanum resin; (ii) “Concrete of *Cistus ladaniferus* (Cistaceae) obtained from stems and leaves by organic solvents extraction”; (iii) “Resinoid of *Cistus ladaniferus* (Cistaceae) obtained from labdanum gum by ethanol extraction” which is in fact an ethanol labdanum absolute. All these registered substances are reported to have no physical, environmental, and human health hazards which include a complete toxicological assessment. However, further toxicological and/or skin irritation tests may be necessary to ensure the safety of using labdanum resin, or its flavonoid and terpenoid fractions, as a whole or in part as cosmetic ingredients, in order to guarantee the complete consumer safety.

## 3. Materials and Methods

### 3.1. Labdanum Absolute

Herbaceous/semiwoody terminal leafy stems (10–30 cm) were collected from a *Cistus ladanifer* subsp. *ladanifer* shrubland in Penha Garcia, Portugal (GPS: N 40°1′43.4″ W 6°59′34.8″), in August 2018. Plant material was stored fresh in the freezer (−20 °C) until use. Subspecies was identified using “Flora Ibérica” by Demoly and Montserrat [2] and two exemplars were deposited in the herbarium collection of Escola Superior Agrária de Castelo Branco, Portugal, under the code 004 ESACBA1 CIS CIS LAD 02.

Labdanum was extracted based on the method described by Burguer [6]. Twenty-five grams of plant material was extracted in 200 mL of Na_2_CO_3_ aqueous solution (25 g/L), for 1 h at 60 °C (water bath), inside a flask. The solution was filtered through a stainless-steel sieve (mesh no. 140, 106 µm aperture). After cooling, H_2_SO_4_ solution (5 M) was added until pH 2. The precipitated resin was separated from the supernatant by centrifugation, at 3030× *g* for 15 min (Mega Star 600R, VWR), and finally freeze-dried.

Labdanum absolute was obtained by removing “waxes” from the labdanum resin based on the method described by Vogt, et al. [11] and used in recent works [3,10,12]. Briefly, one gram of resin, maximum, was dissolved in 20 mL methanol using an ultrasound bath, left at −20 °C overnight, and then centrifuged at 3030× *g* at −5 °C for 15 min. The procedure was repeated three times, and the supernatants were joined. Three independent extractions were conducted.

### 3.2. Diterpenoid and Flavonoid Fractions

Labdanum absolute was subjected to fractionation by molecular-sizing column chromatography as described by Vogt and Gülz [66] and used in recent works [3,10,12]. Absolute was directly eluted on Sephadex^TM^ LH-20 (GE Healthcare Biosciences AB) compacted in a 25 cm long and 1.5 cm width column using methanol as eluent. Fractions of about 2–3 mL were continuously collected and analyzed by thin-layer chromatography using silica gel TLC plates (F254s 0.2 mm 10 × 20 cm) (Merck, Darmstadt, Germany), as described by Vogt and Gülz [66]. Toluene: ethyl acetate (9:1) was used as eluent and, after drying, plates were visualized at 312 nm using a transilluminator to detect flavonoid bands. To detect diterpene bands, plates were sprayed with 10 M H_2_SO_4_ solution and heated at 100 °C for 10 min in a ventilated chamber. Purple/red bands were assigned to the diterpenes and yellow bands to the flavonoids because they matched the bands visualized at 312 nm. Fractions presenting purple bands were joined as the diterpene fraction (Dit) and fractions presenting flavonoid bands were joined as the flavonoid fraction (Flv). This procedure was conducted for the three independent labdanum absolutes.

### 3.3. Chemical Characterization of Absolute and Fractions

Labdanum absolute and fractions were characterized qualitatively by UPLC-DAD-ESI-MS (Nexera X2 system; LC-30AD pump, Shimadzu). Chromatographic separation and analysis were performed, using a Kinetex C18 column (150 mm × 4.6 mm, 2.7 µm particle size) from Phenomenex (Torrance, CA, USA, EUA). The mobile phase comprised solvent A (ultrapure water with 0.1% formic acid) and solvent B (acetonitrile with 0.1% formic acid). Separation was carried out at 0.5 mL/min flow rate, 30 °C oven temperature, and at the following elution gradient: 0 min, 20% solvent B; 60 min, 100% solvent B; 65 min, 100% solvent B; 67 min, 20% solvent B; 70 min, 20% solvent B. Data were acquired using DAD (190–700 nm) and ESI-MS detection (positive and negative mode, *m*/*z* ions 100–2000, interface 4.5 kV, interface temperature 350 °C, desolvation line temperature 250 °C, heat block 200 °C, 1.5 L/min nebulizing gas, 20 L/min drying gas (Nexera X2 SPD-M30A + LCMS 2020, Shimadzu). Labdanum absolutes were injected at the following concentrations: Absolute and Dit: 0.266 mg/mL; Flv: 0.066 mg/mL. The injection volume was 20 µL. Compounds were identified based on their pseudo-molecular ions and literature comparison.

### 3.4. UV Radiation Absorption

Spectrophotometric SPF (sun protection factor) was determined by the method described by Gaweł-Bęben et al. (2020). UV absorbance scan (200–400 nm) of the labdanum absolute and fractions was measured on a spectrophotometer in a 0.5 cm quartz cell using proper dilutions to obtain absorbance values below 1. UV absorbance scan was read in duplicate for each extract replicate and methanol was used as reference. Absorbance values for 100 µg/mL solutions were read at 290, 295, 300, 305, 310, 315, 320 nm, and SPF was calculated by the Mansur Equation (1) (Mansur et al., 1986):

(1) $$SPF=CF\times \overset{320}{\underset{290}{\sum }}EE\left(\lambda \right)\times I\left(\lambda \right)\times Abs\left(\lambda \right)$$

where *EE* (erythemal effect spectrum) × *I* (solar intensity spectrum) at a given λ (wavelength) is a normalized constant proposed by Sayre, et al. [67]. CF is a correction factor that was set at 10 for comparison purposes to Gaweł-Bęben, et al. [24] work. In addition, UVB (280–315 nm) and UVA (315–400 nm) total absorbance (arbitrary units, a.u.) of 100 µg/mL solutions were obtained by integration (area) of the UV absorption spectra.

### 3.5. Antioxidant Activity

Stock solutions of labdanum absolute and fractions were prepared to obtain proper colorimetric readings. All methods were performed in duplicate for each extract replicate.

For the Folin–Ciocalteu method, extracts were further diluted (1:10) with distilled water to obtain 5% *v*/*v* methanol in the final reaction mixture, as suggested by Cicco, et al. [68]. Total phenolic content was determined by mixing 200 µL of standard/sample solution, 650 µL of distilled water, 100 µL of Na_2_CO_3_ (7.5% *m*/*v*), and 50 µL of Folin–Ciocalteu reagent. Mixtures were incubated for 2 h, in the dark at room temperature, and absorbance was read at 725 nm. Gallic acid (0.01–0.2 mg/mL) was used as the standard compound and the results were expressed as gallic acid equivalents (GAE, mg) per sample dry weight (mg). 

ABTS radical was prepared by mixing equal volumes of ABTS (7 mM) and K_2_S_2_O_8_ (2.5 mM), and the solution was allowed to react for 16 h in the dark. Then, the radical solution was diluted in acetate buffer (20 mM, pH 4.5) until absorbance of 0.7 ± 0.02 nm at 734 nm. Scavenging activity was determined by mixing 200 µL of radical solution and 20 µL of standard/sample solution prepared in 10% (*v*/*v*) methanol, or acetate buffer (control). Mixtures were left to react for 15 min, in the dark at room temperature, and absorbance was read at 734 nm on a microplate reader. DPPH^•+^ scavenging activity was assessed by mixing 1300 µL of 80 µM DPPH solution, in methanol, with 150 µL of standard/sample solution or methanol (control). Mixtures were left to react in the dark for 30 min and absorbance was read at 517 nm in a spectrophotometer. For ABTS and DPPH methods, absorbance values were transformed in the percentage of inhibition in relation to the controls.

Ferric reducing antioxidant power (FRAP) was assessed by mixing 1500 µL of FRAP reagent (2.5 mL of 10 mM TPTZ in 40 mM HCl and 2.5 mL of 20 mM FeCl_3_.6H_2_O in a 25 mL solution made up with acetate buffer 0.3 M, pH 3.6), 150 µL of methanol and 50 µL of standard/sample solution or methanol (control). Mixtures were allowed to react for 30 min at 37 °C (water bath) in the dark, and absorbance was read at 595 nm using a spectrophotometer. 

For ABTS, DPPH, and FRAP methods Trolox (0.05–0.3 M) was used as standard, and results were expressed as Trolox equivalents (TE, mg) per sample weight (mg). 

### 3.6. Elastase Inhibition Activity

Elastase inhibition assay was performed in a 96-well microplate and absorbance was read using a microplate spectrophotometer. The assay was performed as described in Taghouti, et al. [69]. Labdanum absolute and fractions, tested at 0.5 and 1 mg/mL, were diluted in PBS from stock solutions prepared in DMSO. The final concentration of DMSO was always less than 5% and a control with 5% DMSO was used to exclude solvent-induced enzyme inhibition. Elastase inhibition was assessed by adding 160 µL of Tris-HCl buffer (0.2 mM, pH 8) and 20 µL of N-(methoxysuccinyl)-ala-ala-pro-val-4-nitroanilide (0.8 mM, in 0.2 mM Tris-HCl, pH 8) to 50 µL of the extracts. The mixtures were incubated for 10 min at room temperature, and 20 µL of elastase (0.4 U/mL, in 0.2 M Tris-HCl, pH 8) was added. After incubation for 20 min, at room temperature, absorbance was read at 410 nm. Methanol was used as the positive control. The percentage of inhibition (% inhibition) was calculated according to Equation (2):

(2) $$\%\;inhibition=\frac{\left(Abs_{control}-Abs_{sample}\right)}{Abs_{control}}\times 100$$

where *Abs* is the absorbance value of the control (*Abs_control_*) and of sample (*Abs_sample_*). 

### 3.7. Anti-Inflammatory Activity

Anti-inflammatory activity of labdanum absolute and fractions was evaluated as the inhibition of lipopolysaccharide (LPS)-induced nitric oxide production in Raw 264.7 cells (mouse macrophages, Abelson murine leukemia virus-induced tumor, acquired from Cell Lines Service CLS, Eppelheim, Germany), as previously described by Silva, et al. [28]. Briefly, Raw 264.7 cells were cultured in complete culture media (Dulbecco’s Modified Eagle Media (DMEM), supplemented with 10% (*v*/*v*) fetal bovine serum (FBS), 1 mM L-glutamine, and antibiotics (penicillin 100 U/mL, streptomycin 100 μg/mL) in an incubator (5% CO_2_; 37 °C, controlled humidity). For experiments, cells seeded in 96-well plates (5 × 10^4^ cells/mL; 100 μL per well) were exposed to non-cytotoxic concentrations of labdanum absolutes (prepared in FBS-free culture media) in the presence and in the absence of LPS (1 µg/mL). After a 24 h incubation, 50 µL of supernatant of each well was transferred to a new 96-well microplate, to which 50 µL of Griess reagent, prepared as described by [Silva, et al. [70]], was added, followed by a 15 min incubation in the dark, at room temperature. The absorbance was read at 550 nm, and a sodium nitrite (NaNO_2_) standard curve was used for nitric oxide quantification. Results were expressed as % of control (LPS-stimulated cells not exposed to extracts). The positive control (100% inflammation) and the negative control (0% inflammation) were the LPS-stimulated cells and the cells only exposed to the culture medium, respectively.

### 3.8. Antimicrobial Activity

Reference microorganism strains used in susceptibility tests were Escherichia coli ATCC^®^ 25922TM, Pseudomonas aeruginosa ATCC^®^ 27853TM, Staphylococcus aureus ATCC^®^ 25923TM, and Candida albicans ATCC^®^ 10231TM, acquired from ielab (Alicante, Spain). Labdanum absolute and fractions were prepared at 10 mg/mL directly in culture medium with 10% (*v*/*v*) DMSO. Five 1:2 serial dilutions were conducted for each extract, resulting in five solutions with twice the extract concentration to test and twice the DMSO percentage tolerated (5% v/v, found by a preliminary test with all microorganisms used). In 96-well plates, 50 µL of the extract was mixed with 50 µL of microbial suspension, resulting in 1:2 dilutions, for both. A control was prepared by using 50 µL of culture medium and 50 µL of microbial suspension. Another control was prepared by using 50 µL of culture medium and 50 µL of each sample solution. Müeller-Hinton Broth (OXOID, Basingstoke, United Kingdom) was used for *E. coli*, *P. aeruginosa*, and *S. aureus* and Potato Dextrose Broth (VWR, Leuven, Belgium) was used for *C. albicans*. Strains, from −80°C storage, were grown overnight on Potato Dextrose Agar (HIMEDIA, Nashik, India) or Müeller-Hinton Agar (OXOID, Basingstoke, United Kingdom) and sub-cultured three times before use to prepare the microbial suspensions in 0.9% (*w*/*v*) NaCl saline solution. Bacterial suspensions were adjusted to a 0.5 McFarland standard (bioMérieux, Marcy-L’Étoile, France) and yeast suspensions to a 1.0 standard. From the initial suspension, a 1:100 dilution, in broth medium, was conducted to obtain twice the concentration needed for the susceptibility test. Microplates were incubated for 24 h at 37 °C. Afterward, 5 µL from each well were inoculated on solid medium plates, which were again incubated for an additional 24 h to determine the Minimum Microbicidal Concentration (MMC). Meanwhile, 20 µL of 0.01% (*m*/*v*) resazurin (VWR, Leuven, Belgium) was added to each well of the microplate and then incubated for a further 2 h to determine the Minimum Inhibitory Concentration (MIC). This method was designed based on CLSI M07-A9 Standard and was performed in duplicate for each extract replicate.

### 3.9. Statistical Analysis

Statistical analysis was used to find significant variable effects and significant differences between means of different treatments (*p* < 0.05) and was performed using IBM SPSS Statistics 25 and GraphPad Prism 9 software. Shapiro–Wilk’s test was used to test the normality of data and Levene’s test to test homogeneity of variance. ANOVA *t*-test and a post-hoc Tuckey’s HSD test were used for normal data with homogeneous variance. Welch’s ANOVA, Welch’s t-test, and Dunnet’s T3 post-hoc test were used for normal data with equal variances not assumed.

## 4. Conclusions

Labdanum absolute represents about 70% (*dw*/*dw*) of labdanum resin and is principally composed of labdane-type diterpenes and methylated flavonoids. Labdanum absolute was shown to possess relevant UV protection and anti-inflammatory activities mainly because of the flavonoids fraction. These properties are relevant for skincare cosmetics regarding the damage effects that UV radiation has on the constantly exposed human skin. However, at least within the EU, labdanum resin needs to be included in a list of substances approved to be used as UV filters in cosmetics regulations and if used for its anti-inflammatory activity labdanum resin must be considered a medicinal product and most likely must comply with the regulatory dispositions for medicinal products under a different regulation. Labdanum absolute showed low antioxidant and antimicrobial activity meaning it is not suitable as a cosmetic preservative. Despite that, the extract showed selective and high inhibitory activity against *Staphylococcus aureus*, compared to other cosmetic and skin relevant microorganisms, which is the most common cause of opportunistic skin infections.

## Figures and Tables

**Table 1:** Labdanum resin yield (% FW/DW) in relation to plant material, and labdanum absolute and column chromatography fractions yields (% DW/DW) in relation to labdanum resin (*n* = 3, mean value ± standard deviation).

Resin	Absolute	Dit	Flv	Remainder
7.44 ± 0.41	73.3 ± 0.9	74.2 ± 1.8	15.6 ± 4.6	10.1 ± 2.9

Abbreviations: Dit, Diterpene fraction; Flv, Flavonoid fraction.

**Table 2:** UPLC-DAD-ESI-MS peaks in the chromatograms of labdanum absolute and fractions. Considering the four detection signals (absorbance at 250 and 350 nm and total ion count at positive and negative modes), +: presence and ++: major peak. Peak order number, retention time (min), ESI ions weight (*m*/*z*^−^ and *m*/*z*^+^), literature compound match (considering pseudo-molecular ions [M-H]^−^ and [M + H]^+^), matched compound molecular weight, and literature references of the matched compound are presented. ESI ions and matched compound weight numbers rounded down. Highlighted with light grey background: matched flavonoids; with dark grey background: diterpenoids; and no background colour: no match with literature.

Peak	Retention Time (min)	ESI Ions		Literature Compounds Match					
		[M-H]^−^ (*m/z*)	[M + H]^+^(*m/z*)	Absolute	Dit	Flv	Compound	MW (g/mol)	Reference
1	16.8	269	271	+		+	Apigenin	270	[10,36,38,39,40]
2	18.4	299	301	++		++	Kaempferol-3-methylether (isokaempferide)	300	[10,36,38,39,40]
3	24.7	283	285	+		+	Apigenin-4′-methylether (acacetin)	284	[10,36,38,39,40]
4	24.9	283	285	+		+	Apigenin-7-methylether (genkwanin)	284	[9,10,36,38,39,40]
5	26.6	313	315	++		++	Kaempferol-dimethylether (3,7, jaranol, or 3,4′)	314	[10,36,38,39,40,41,42]
6	28.6	335	337		+		-	(336)	
7	30.9	379	363 (292/303/321)		+		-	(380)	
8	32.0	381	-		+		-	(382)	
9	32.2	-	-		+		-		
10	33.1	319	321		+		Oxo-labdenoic acid	320	[7,8,9,12,37,43]
11	33.5	309 (379)	-		+		Labdandiol (p.e. 8,15)	310	[9,44,45]
12	33.9	-	299	+		+	Apigenin-7,4′-dimethylether	298	[9,36,38]
13	35.4	319	321	+	++		Oxo-labdenoic acid	320	[3,7,8,9,12,37]
14	35.7	-	329	+		+	Kaempferol-3,7,4′-trimethylether (methyljaranol)	328	[9,36,38,46]
15	37.4	323	-		++		Labdanolic acid	324	[6,7,8,9,37,44,47]
16	37.9	-	307		+		Labdenoic acid (8(17) ladenic, 7 cativic acids)	306	[6,7,44,48]
17	38.4	-	257/351 (375)	+			8α-methoxy-labdan-15-oic acid	256	[9,37,44]
18	39.6	-	257		+		-	(256)	
19	40.1	371	338 (398/241)		+		-	(372)	
20	42.3	-	456 (393)	++			-	(455)	
21	43.6	381/433	335 (376/435/269)	+			-	(382/434)	
22	44.4	-	384	+			-	(383)	
23	45.9	-	335 (376)	++	+		-	(334)	
24	47.1	-	271		+		-	(270)	
25	48.1	-	398 (165)		+		-	(397)	

Abbreviations: Dit, Diterpene fraction; Flv, Flavonoid fraction.

**Table 3:** Spectrophotometric Sun Protection Factor (SPF), and UVB (280–315 nm) and UVA (315–400 nm) total absorbance (area under the curve, in a.u.) of 100 µg/mL solutions of labdanum absolute and diterpene (Dit) and flavonoid (Flv) fraction (*n* = 3, mean value ± standard deviation).

		Total Absorbance (a.u.)	
Extract	SPF	UVB	UVA
Absolute	4.98 ± 0.19 ^b^	9.05 ± 0.36 ^b^	16.7 ± 0.6 ^b^
Dit	0.736 ± 0.015 ^c^	1.44 ± 0.02 ^c^	1.67 ± 0.03 ^c^
Flv	13.0 ± 1.4 ^a^	22.0 ± 2.3 ^a^	47.1 ± 5.3 ^a^

Note: Different coefficient letters mean statistical differences (*p* < 0.05).

**Table 4:** Folin–Ciocalteu test, 1,1-diphenyl-2-picrylhydrazyl free radical scavenging activity (DPPH), Ferric ion Reducing Antioxidant Power (FRAP), and 2,2′-azinobis(3-ethylbenzothiazoline-6-sulphonic acid free radical scavenging activity (ABTS) of labdanum absolutes and fractions, expressed as Gallic acid (GAE) or as Trolox (TE) equivalents, in the dried extract (Ext) (*n* = 3, mean values ± standard deviation).

Extract	Folin–Ciocalteu	DPPH	FRAP	ABTS
	(mgGAE/mgExt)	(mgTE/mgExt)		
Absolute	0.128 ± 0.015 ^a,b,c^	0.031 ± 0.008 ^a,b,c^	0.038 ± 0.008 ^a,b,c^	0.142 ± 0.017 ^a,b,c^
Flv	0.203 ± 0.044 ^a,b^	0.054 ± 0.005 ^a,b^	0.049 ± 0.004 ^a,b^	0.379 ± 0.039 ^a,b^
Dit	0.011 ± 0.001 ^b,c^	0.005 ± 0.001 ^b,c^	0.020 ± 0.003 ^b,c^	0.010 ± 0.003 ^b,c^

Note: Different coefficient letters mean statistical differences between extracts for each method (*p* < 0.05).

**Table 5:** Inhibition of elastase activity (% of control) by the labdanum absolute and fractions at concentrations of 1 and 0.5 mg/mL (*n* = 3, average value ± standard deviation).

	Elastase Inhibition (% of Control)	
Extract	1 mg/mL	0.5 mg/mL
Absolute	22.074 ± 0.292 *	n.a.
Dit	n.a.	-
Flv	13.711 ± 0.313 *	n.a.

Note: “n.a.”: no activity considered when inhibition mean values of the extracts were not statistically different from the mean value of the negative control. * Means values statistically different (*p* < 0.05). “-”: not tested.

**Table 6:** Antimicrobial activity: Minimum Inhibitory Concentration (MIC) and Minimum Microbicidal Concentration (MMC), of labdanum absolute and fractions in relation to selected microorganisms (n = 3, mode value).

	*E. coli*	*P. aeruginosa*	*S. aureus*		*C. albicans*
Extracts			MIC	MMC	
Absolute	n.a.	n.a.	1.2	n.a.	n.a.
Flv	n.a.	n.a.	≤0.3	2.5	n.a.
Dit	n.a.	n.a.	≤0.3	2.5	n.a.

Note: MIC and MMC values in mg/mL. “n.a.”: no activity in relation to the control. *E. coli* ATCC 25922, *P. aeruginosa* ATCC 27853, *S. aureus* ATCC 25923, *C. albicans* ATCC 10231.

## Data Availability

Not applicable.

## Supplementary Materials

The following supporting information can be downloaded at: https://www.mdpi.com/article/10.3390/plants11111477/s1, Figure S1.1. DAD (250 and 350 nm, absolute intensity) and MS (TIC, *m*/*z*^−^ and *m/z*^+^, relative intensity) chromatograms were obtained in the UPLC-DAD-ESI-MS analysis of labdanum absolute. Numbers represent a peak at a retention time as presented in Table 2. Figure S1.2. DAD (250 and 350 nm, absolute intensity) and MS (TIC, *m/z*^−^ and *m/z*^+^, relative intensity) chromatograms were obtained in the UPLC-DAD-ESI-MS analysis of diterpenoid fraction from labdanum absolute. Numbers represent a peak at a retention time as presented in Table 2. Figure S1.3. DAD (250 and 350 nm, absolute intensity) and MS (TIC, *m/z*^−^ and *m/z*^+^, relative intensity) chromatograms were obtained in the UPLC-DAD-ESI-MS analysis of “flavonoid” fraction from labdanum absolute. Numbers represent a peak at a retention time as presented in Table 2. Figure S2. RAW 264.7 (murine macrophage) cells viability, as a percentage of the control.
