# Supplementary material for: Labdanum Resin from Cistus ladanifer L.: A Natural and Sustainable Ingredient for Skin Care Cosmetics with Relevant Cosmeceutical Bioactivities
Source: Plants (Basel). 2022 May 31;11(11):1477. doi: 10.3390/plants11111477 (PMC9183103; doi:10.3390/plants11111477)
Supplement: Supplementary file 1 [file plants-11-01477-s001.zip › plants-1742627-supplementary.pdf]

Supplementary material

Labdanum resin from *Cistus ladanifer* L.: a natural and sustainable ingredient for skin care cosmetics with relevant cosmeceutical bioactivities

David F. Frazão [1, 2], Carlos Martins-Gomes [1], Jan L. Steck [3], Judith Keller [3], Fernanda Delgado [2, 4], José C. Gonçalves [2, 4], Mirko Bunzel [3], Cristina M. B. S. Pintado [2, 4], Teresa Sosa Díaz [5], Amélia M. Silva [1, 6,*]

[1] Center for Research and Technology of Agro-Environmental and Biological Sciences (CITAB-UTAD), University of Trás-os-Montes e Alto Douro (UTAD), Quinta de Prados, 5001-801 Vila Real, Portugal; davidmfrazao@gmail.com (D.F.F.), camgomes@utad.pt (C.M.G.)

[2] Plant Biotechnology Center of Beira Interior (CBPBI), Quinta da Senhora de Mércules, Apartado 119, 6001-909 Castelo Branco, Portugal; jcgoncalves@ipcb.pt (J.C.G.),

[3] Department of Food Chemistry and Phytochemistry, Institute of Applied Biosciences, Karlsruhe Institute of Technology (KIT), Adenauerring 20a, Building 50.41, 76131 Karlsruhe, Germany; jan.steck@kit.edu (J.L.S.), judith.keller@kit.edu (J.K.), mirko.bunzel@kit.edu (M.B.)

[4] Polytechnic Institute of Castelo Branco - School of Agriculture (IPCB-ESA), Quinta da Senhora de Mércules, 6001-909 Castelo Branco, Portugal; fdelgado@ipcb.pt (F.D.), cpintado@ipcb.pt (C.M.B.S.P.)

[5] Department of Plant Biology, Ecology and Earth Sciences, Faculty of Science, University of Extremadura, 06006 Badajoz, Spain; tesosa@unex.es (T.S.D.)

[6] Department of Biology and Environment, School of Life Sciences and Environment, UTAD, Quinta de Prados; 5001-801 Vila Real, Portugal; amsilva@utad.pt (A.M.S.)

* Correspondence: amsilva@utad.pt (A.M.S.); Tel.: +351 259 350 921

Supplementary Figure S1

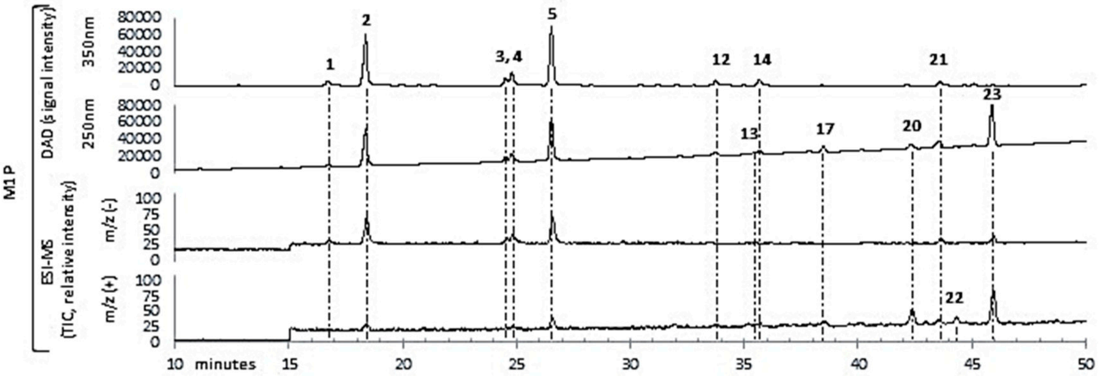

Figure S1.1. DAD (250 and 350 nm, absolute intensity) and MS (TIC, m/z^- and m/z^+, relative intensity) chromatograms obtained in the UPLC-DAD-ESI-MS analysis of labdanum absolute. Numbers represent a peak at a retention time as presented in Table 2.

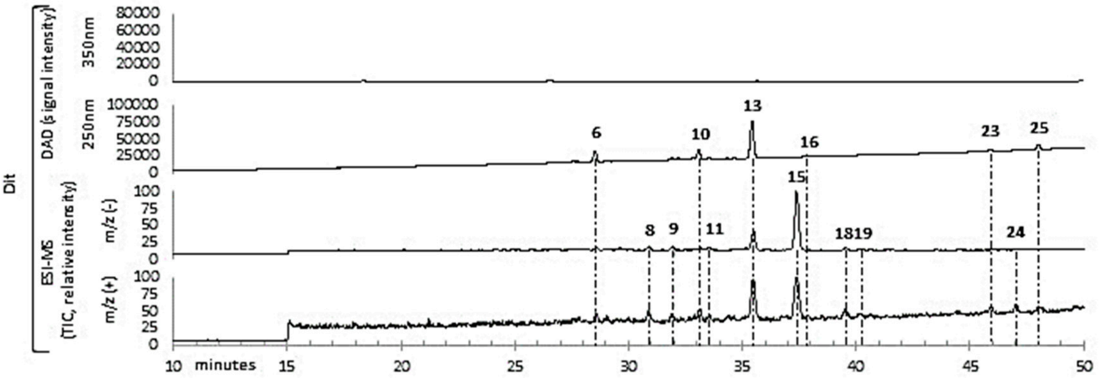

Figure S1.2. DAD (250 and 350 nm, absolute intensity) and MS (TIC, m/z^- and m/z^+, relative intensity) chromatograms obtained in the UPLC-DAD-ESI-MS analysis of diterpenoid fraction from labdanum absolute. Numbers represent a peak at a retention time as presented in Table 2.

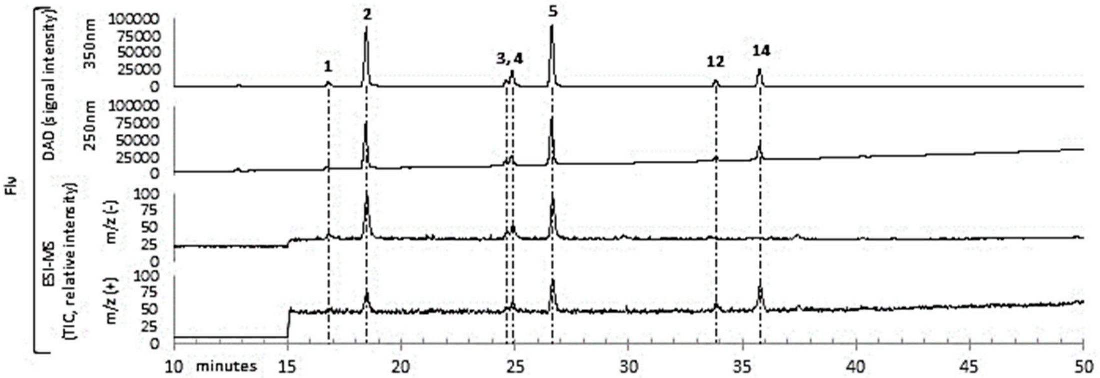

Figure S1.3. DAD (250 and 350 nm, absolute intensity) and MS (TIC, m/z^- and m/z^+, relative intensity) chromatograms obtained in the UPLC-DAD-ESI-MS analysis of "flavonoid" fraction from labdanum absolute. Numbers represent a peak at a retention time as presented in Table 2.

Supplementary Figure S2

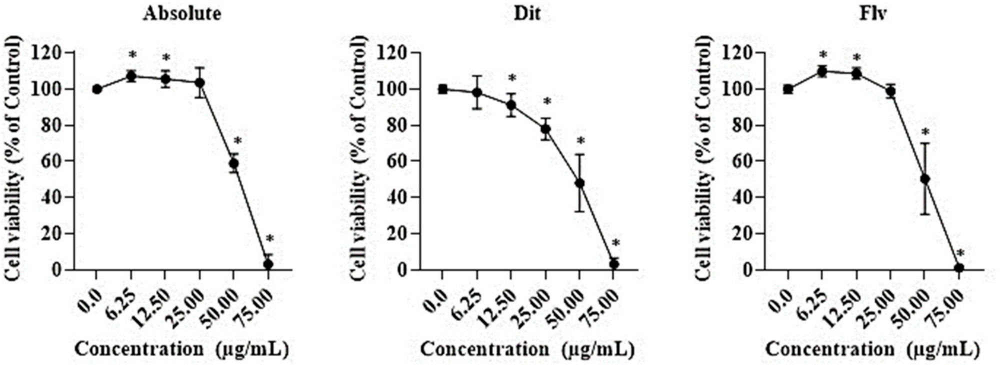

Figure S2. RAW 264.7 (murine macrophage) cells viability, as percentage of control (cells not exposed to the compounds, denoted as 0.00 µg/mL), of labdanum absolute and its fractions at concentrations between 6.25 and 100 µg/mL (mean values ± S.D.). Symbol (*) mean significant difference (α = 0.05) in relation to the control by the post-hoc Tuckey's test.
